# A Game-Theoretic Model of Interactions between Hibiscus Latent Singapore Virus and Tobacco Mosaic Virus

**DOI:** 10.1371/journal.pone.0037007

**Published:** 2012-05-18

**Authors:** Zibo Chen, Jackie Yen Tan, Yi Wen, Shengniao Niu, Sek-Man Wong

**Affiliations:** 1 Department of Biological Sciences, National University of Singapore, Singapore, Singapore; 2 Undergraduate Research Opportunities in Science (UROPS), National University of Singapore, Singapore, Singapore; 3 University Scholars Programme (USP), National University of Singapore, Singapore, Singapore; 4 Temasek Life Sciences Laboratory, National University of Singapore, Singapore, Singapore; Central China Normal University, China

## Abstract

Mixed virus infections in plants are common in nature and their interactions affecting host plants would depend mainly on plant species, virus strains, the order of infection and initial amount of inoculum. Hence, the prediction of outcome of virus competition in plants is not easy. In this study, we applied evolutionary game theory to model the interactions between Hibiscus latent Singapore virus (HLSV) and Tobacco mosaic virus (TMV) in *Nicotiana benthamiana* under co-infection in a plant host. The accumulation of viral RNA was quantified using qPCR at 1, 2 and 8 days post infection (dpi), and two different methods were employed to predict the dominating virus. TMV was predicted to dominate the game in the long run and this prediction was confirmed by both qRT-PCR at 8 dpi and the death of co-infected plants after 15 dpi. In addition, we validated our model by using data reported in the literature. Ten out of fourteen reported co-infection outcomes agreed with our predictions. Explanations were given for the four interactions that did not agree with our model. Hence, it serves as a valuable tool in making long term predictions using short term data obtained in virus co-infections.

## Introduction

Viral infections are common in plants and it is not unusual for more than one virus species to infect the same host. These mixed infections usually generate effects that are not observed in single infections as plants are often found to be co-infected with multiple viruses [1]. The synergy ranges from mutual increase in virus titre, asymmetric increase in titre of one virus, while no change for another virus [2]–[3], to no change for one virus but asymmetric decrease in titre of the other virus [4]. There have been cases of mutual interference, which causes a decrease in both viruses [5]. Various strategies such as genetically modifying crops for virus resistance have been explored to minimize losses from infections caused by viruses such as Tobacco mosaic virus (TMV). With the current uncertainty on genetically modified plants as a solution against viral infections [6]–[7], exploring the usage of co-infecting viruses to cross-protect as an alternative solution to counter virulent plant viruses seems to be a viable alternative. Cross protection describes the phenomenon which an infection of a mild strain virus protects plants against subsequent infection from the severe strain of a closely related virus [8]. When two unknown viruses interact in the same host, the outcome of the interaction is often unpredictable. Thus, there is a need to conduct co-infection experiments to identify the dominant virus.

TMV is a tobamovirus with a broad host range [9]. It causes necrosis in *Nicotiana benthamiana* Domin which leads to plant death. Hibiscus latent Singapore virus (HLSV) is a new member of *Tobamovirus* and unlike TMV, it does not cause necrosis in its host. Instead, it induces mild symptoms such as slight crinkling of leaves in *N. benthamiana* [10]. It has been observed that in superinoculation experiments, *N. benthamiana* can be cross-protected against TMV by HLSV (unpublished data). However, co-inoculation of both viruses onto the same plant has not been carried out. Here we applied evolutionary game theory to examine the interactions between these two viruses in co-infection.

Evolutionary game theory was formulated to analyze a population of interacting players, whose fitness are frequency dependent [11]. It is a two-player, non-zero sum, non-cooperative game with finite number of strategies and symmetric pay-offs [12]. Unlike a standard non-cooperative game in which each player knows all the details of the game and plays rationally, evolutionary game theory describes a game in which players are ‘pre-programmed’ to some strategies. In other words, the strategies employed by players are genetically determined [13]. Prior to replication, an individual virus randomly encounters another virus, thus engaging each other in an interaction. This interaction results in a pay-off, which is the change in fitness in the said individual. The expected number of surviving progenies is thus proportional to the individual’s fitness. The motivation behind modelling the two populations’ interactions using evolutionary game theory is to predict long term interaction outcome using short term data. In other words, it is the period before symptoms of virus infection appear. Conversely, the words ‘long term’ is defined here as the time needed to observe the symptoms of virus infection and to identify the dominating virus, either qualitatively or quantitatively. Hence the period of ‘long term’ is relative and it will depend on the particular combinations of host plants and viruses. ‘Short term’ refers to the minimum time needed to predict the interaction outcome that agrees with the long term data.

In a game involving two players and two strategies, a pay-off matrix is used to model their interactions (Table 1).

**Table 1:** The pay-off matrix for the interactions between a Player and an Opponent.

Player	Opponent	
	A	B
A	a	b
B	c	d

Each player has a different fitness depending on which other individual it has interacted (played) with. Player A has a fitness of *a* if playing against Player A, a fitness of *b* against Player B. On the other hand, Player B has a fitness of *c* against Player A and a fitness of *d* against Player B. Under the assumption of a well-mixed population where players encounter each other randomly, there are four outcomes which depend on the relative fitness between the two players resulting from their interactions. Player A dominates the game if *a*>*c* and *b*>*d*. Similarly, player B dominates the game if *a*<*c* and *b*<*d*. In any mixed population, it will converge towards a homogenous population containing the dominant player. The fitness of the dominant player is a result of the interactions between both players and it is relatively higher than the opponent. However, when there is no clear dominance, i.e. when *a*>*c* and *b*<*d*, player A plays better against player A, but not against player B and vice versa. Both strategies are in strict Nash equilibrium [14], the well-mixed population will converge to either one of the players, depending on which strategy becomes established first [12]. When *a*<*c* and *b*>*d*, both strategies are equally competitive. The well mixed population will remain heterogeneous as it is more advantageous for both players to interact with opponents, as compared to a homogenous population.

In the context of a co-infection, the viruses are both the players and the strategies. By virtue of being a virus itself is a strategy because each species of viruses possesses a set of behaviours such as replication, expression of coat protein, virion assembly, virus movement, etc, which differentiates it from other virus species/players. Application of game theory to the interactions between plant viruses during mixed infection has yielded interesting insights into the synergism between different species of viruses [15]; but the model needs to be generalized in order to have a broader range of applications. We achieve this by identifying the evolutionarily stable strategy (ESS) in their interactions. ESS is a strategy which cannot be dominated by any other alternative strategies in a game, leading to equilibrium in a game. By identifying the ESS in a particular interaction between two viruses, we are able to predict the long term outcome of viral interactions using short term data.

In this study, we investigated the virus accumulation in *N. benthamiana* plants infected with HLSV and/or TMV under co-infection. The collected short term data was then used to model their interactions and to predict their long term interaction outcome.

## Results and Discussion

### Changes of Virus Accumulation in Single and Mixed Infections

**Figure 1:**
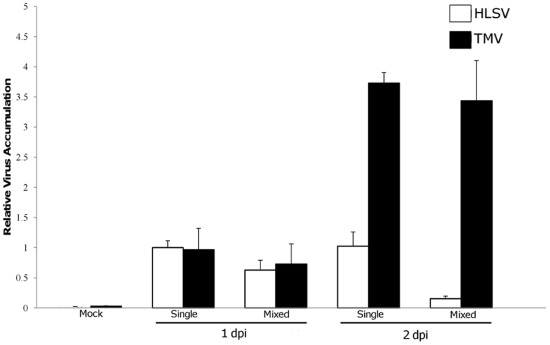
Relative RNA amount of HLSV and TMV 1 dpi and 2 dpi. The amount of viral RNA was calculated through quantitative PCR.

To compare the fitness of the two viruses under single and mixed infections, we computed the relative virus accumulation. It was computed as the ratio of the amount of viral RNA accumulated in each infection compared with the values obtained for HLSV in single infections. The relative means ± SEM of viral RNA accumulation for each virus at 1 and 2 dpi, respectively, with single and mixed infections, were compared (Fig. 1), using the 1 dpi viral accumulation of HLSV as the baseline. At 1 dpi, HLSV RNA accumulated at 1.000±0.113, while TMV RNA accumulated at 0.967±0.354 (Mann-Whitney test, P = 0.115). In mixed infections, HLSV accumulation decreased to 0.629±0.162, a 47.1% decrease (Mann-Whitney test, P = 0.045), while TMV decreased to 0.730±0.335, a 24.5% decrease (Mann-Whitney test, P = 0.05), suggesting a competition between the two viruses.

At 2 dpi, HLSV accumulated at 0.983±0.237, a 1.7% decrease but the decrease was not statistically significant (Mann-Whitney test, P = 0.64). HLSV takes time to replicate and as such the difference in viral RNA accumulation between 1 and 2 dpi was relatively small. On the other hand, TMV increased by 241% to 3.299±0.176 (Mann-Whitney test, P = 0.017). In mixed infections, HLSV accumulation in 2 dpi decreased to 0.146±0.045, an 85.1% decrease (Mann-Whitney test, P<0.002). Similarly, TMV level in mixed infections experienced a 1.85% decrease, to 3.238±0.664 (Mann-Whitney, P = 0.79). The presence of HLSV lowers the relative amount of TMV, as observed on the first day.

### Prediction of Long Term Outcomes Using Short Term Data

The relative accumulation of the two viruses allows us to examine their relative fitness. We have constructed the pay-off matrix by arranging the relative accumulation of virus in single and mixed infections for both days (Table 2).

**Table 2:** The pay-off matrix for 1 dpi and 2 dpi between HLSV and TMV.

	Opponent	
1 dpi	HLSV	TMV
HLSV	1.000±0.113	0.629±0.162
TMV	0.730±0.335	0.967±0.354
2 dpi		
HLSV	0.983±0.237	0.146±0.045
TMV	3.238±0.664	3.299±0.176

The table is constructed using values from qRT-PCR.

TMV had a higher fitness when competing against its own population (*d* = 0.967±0.354>*c* = 0.730±0.335) but a lower fitness when competing against HLSV (*a* = 1.000±0.113>*b* = 0.629±0.162). Modelling with one of the four scenarios described above, the two viruses were in Nash equilibrium; both viruses had equally competitive strategies and were able to overcome each other, depending on which strategy was established first. One of the prediction methods is to calculate the expected pay-off values, or fitness of each player [12]. In a well mixed population with equal amount of HLSV and TMV, the expected pay-off of HLSV is
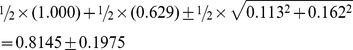
Similarly, the expected pay-off for TMV is calculated to be 0.8485±0.2437. As the pay-off of TMV slightly exceeded that of HLSV, it could be speculated that TMV began to dominate the game at 1 dpi with a small selection advantage and it was highly likely that TMV would dominate the game in the long run.

**Figure 2:**
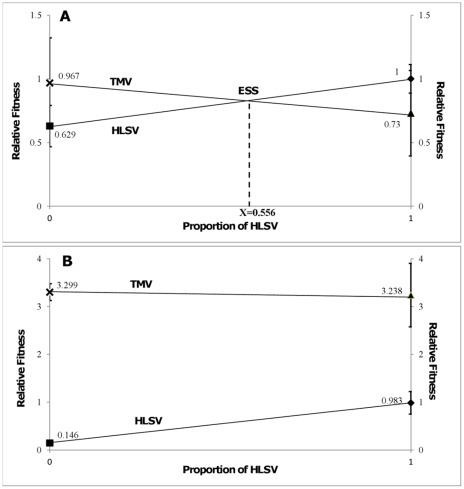
Graphical representations of an alternate method to determine the evolutionary stable strategy (ESS). The graph was constructed using data obtained from 1 dpi (a) and 2 dpi (b). The horizontal axis represents the proportion of HLSV in the population. The left vertical axis shows the relative fitness of TMV and HLSV when no HLSV was present in the population. The right vertical axis shows the relative fitness of TMV and HLSV when all the other competitors were HLSV. The lines connect the fitness of either viruses under both single- and mixed-infections, and the x-value of their intersection represents the proportion of HLSV in the ESS.

An alternative way of making predictions is by finding ESS. Both viruses would reach a strict Nash equilibrium if ESS was played. At 1 dpi, the ESS was a mixed strategy; it was a combination of each pure strategy (both HLSV and TMV). Figure 2a is constructed to show the ESS estimation [12], in which the fitness of each virus under both single and mixed infections are connected by the two lines. From Figure 2a, the two y-axes correspond to the type of viruses present in the interaction. TMV was assigned on the left and HLSV on the right. Taking HLSV as a focal point, when the x-value (proportion of HLSV) is zero, it means the competitor is purely TMV, i.e. proportion of HLSV as a competitor is zero. Hence, TMV would have a relative fitness of 0.967 while HLSV has a relative fitness of 0.629. When the x-value is 1, it means the second player in the interaction is purely HLSV. As such, HLSV would have the baseline value of 1 while TMV has a relative fitness of 0.73.

The x-value (0.556) of the intersection between two lines represents the proportion of HLSV in an ESS. It is estimated that in a population consisting of 55.6% HLSV and 44.4% TMV, both viruses will have the same fitness value. However, such condition is difficult to achieve in actual experiments, because viruses cannot be perfectly mixed in a biological system. Also, any fluctuations of initial virus amount could lead to oscillations and chaotic behaviours [16]. As the initial percentage of TMV in the population is 50% which is greater than its proportion in ESS (44.4%), it can be predicted that TMV would break the equilibrium and dominate the game. Thus, determination of ESS provides us with an alternate tool to predict long term outcomes using short term data.

The 2 dpi data was used to confirm the predictions made based on 1 dpi data. At 2 dpi, the pay-off values differed significantly (Table 2). TMV always showed higher fitness when competing against its own population (*d* = 3.299±0.176>*c* = 3.238±0.664) or against HLSV (*c* = 3.238±0.664> *a* = 0.983±0.237). The expected pay-off for TMV was calculated to be 3.3135±0.3435, being significantly higher than that of HLSV (0.5645±0.1206). Using Straffin chart as an alternative method (Figure 2b), the line connecting fitness of TMV is above that of HLSV and they do not intersect within the range of [0,1]; hence TMV itself became the ESS. It can be predicted that TMV would dominate the game in the long run and the TMV population could no longer be invaded by HLSV, which is consistent with the prediction made from data obtained at 1 dpi.

The predictions made using either 1 or 2 dpi data were consistent with the long term interaction outcome, namely all the co-infected plants died after 15 dpi due to systemic invasion of TMV. Also, the qRT-PCR data collected at 8 dpi revealed that the viral accumulation of TMV was more than a 100 times higher than that of HLSV in mixed infection (data not shown). Hence, these two methods proved to be useful in making long term predictions using short term data.

### Validation of Model

To investigate the validity of our proposed model, we conducted a literature search of scientific papers published on plant virus co-infection and characterization of interactions of two viruses in a single host plant. A total of 14 virus-virus interactions out of 9 papers that contained data on the short term and long term quantifications of the viruses were investigated [2], [15], [17]–[23]. The earliest data reported in those papers was taken as short term data, while the latest data as long term data. The short term data was subjected to our model and the predicted dominance and the reported dominance of the viruses in co-infections were compared for validation (Table 3). The corresponding Straffin charts which depict the extracted data from the above 14 interactions are presented in supplementary materials (Figures S1, S2, S3, S4, S5, S6, S7, S8, S9, S10, S11, S12, S13, and S14).

**Table 3:** A summary of the extracted results of the 9 papers.

Virus 1	Virus 2	Host	PredictedDominance	ReportedDominance	Existenceof ESS	References
Maize chlorotic mottlemachlovirus (MCMV)	Wheat streak mosaicrymovirus (WSMV)	N84Ht corn	MCMV	MCMV	No	Scheets (1998)
Zucchini yellow mosaic virus (ZYMV)	Cucumber mosaic virus (CMV)	Zucchini(cv. Escandarani)	CMV	CMV	Yes	Fattouh (2003)
Sweet potato chloroticstunt virus (SPCSV)	Sweet potato featherymottle virus common strain(SPFMV-C)	Sweet potato	SPCSV	SPCSV	No	Kokkinos and Clark (2006)
Sweet potato chloroticstunt virus (SPCSV)	Sweet potato virus G (SPVG)	Sweet potato	SPCSV	SPCSV	No	ibid.
Cucumber mosaic virus(CMV)	Pepper mottle virus(PepMoV)	Capsicum annuum(cv. Early Calwonder)	PepMoV	PepMoV	Yes	Murphy and Bowen (2006)
Zucchini yellow mosaicvirus (ZYMV-SD)	Cucumber mosaic virus(CMV-Fny)	Cucumber	ZYMV-SD	CMV-Fny	No	Zeng *et al* (2007)
Zucchini yellow mosaicvirus (ZYMV-SD)	Cucumber mosaic virus(CMV-Fny)	Bottle gourd	CMV-Fny	CMV-Fny	Yes	ibid.
Tomato chlorosis virus(ToCV)	Tomato infectious chlorosisvirus (TICV)	Nicotiana benthamiana	ToCV	TiCV	Yes	Wintermantel *et al* (2008)
Tomato chlorosis virus(ToCV)	Tomato infectious chlorosisvirus (TICV)	Physalis wrightii	ToCV	ToCV	Yes	ibid.
Turnip mosaic virus (TuMV)	Cauliflower mosaiccaulimovirus (CaMV)	Arabidopsis thaliana	TuMV	TuMV	No	Martín and Elena (2009)
Tomato rugose mosaic virus (ToRMV)	Tomato yellow spot virus (ToYSV)	Nicotiana benthamiana	ToYSV	ToYSV	No	Alves-Júnior *et al* (2009)
Tomato rugose mosaic virus (ToRMV)	Tomato yellow spot virus (ToYSV)	Tomato	ToYSV	ToYSV	No	ibid.
Triticum mosaic virus (TriMV)	Wheat streak mosaic virus (WSMV)	Wheat(cv. Tomahawk)	WSMV	TriMV	Yes	Tatineni *et al* (2010)
Triticum mosaic virus (TriMV)	Wheat streak mosaic virus (WSMV)	Wheat(cv. Arapahoe)	WSMV	TriMV	No	ibid.

Evolutionary game theory has been used to study the interactions between two different groups of viruses [15], our model generalizes its application to either genetically closely related or unrelated viruses. Our proposed model was able to predict the long term results of the virus co-infections using short term data in 10 interactions out of the 14 co-infections. The 4 interactions that did not fit our model were Zucchini yellow mosaic virus (ZYMV-SD) and Cucumber mosaic virus (CMV-Fny) in cucumber [2], Tomato chlorosis virus (ToCV) and Tomato infectious chlorosis virus (TiCV) in *N. benthamiana* [21], Wheat streak mosaic virus (WSMV) and Triticum mosaic virus (TriMV) in wheat cultivars Tomahawk and Arapahoe [23]. We predicted that ZYMV-SD would dominate in cucumber plants, but CMV-Fny was reported to dominate instead. We had difficulty extracting data from the paper as the baseline for single virus level is zero. Furthermore, it was probable that 7 dpi was not long enough to be indicative of the final interaction outcome for them. As for the interaction outcome between ToCV and TiCV in *N. benthamiana*, we were unable to determine the upper line in the Straffin chart because of the relatively large error bars (Figure S8). In the co-infection of WSMV and TriMV in two different cultivars of wheat, both wheat cultivars possess temperature-dependent resistance against WSMV but not TriMV [24]. Since our model assumed no effect from host plants on virus competition, host resistance allowed TriMV to gain an unfair advantage over WSMV in the long term. Thus, it explains the observed discrepancy between the predicted and reported data.

In this experiment, we assume that the players in the game do not switch their strategies to gain an advantage over their opponents. However, there are cases where a player “switches” strategy to gain an advantage over the other players in order to dominate the game, e.g. escape from a Prisoner’s Dilemma through clonal selection of fitter progenies [25] and production of more RNA under stress [26]. Thus, an alternative interpretation on the results is that TMV has essentially ‘defected’, opting to cheat and play an unfair game against HLSV by disrupting the ESS. Further *in vitro* evolution studies of viruses may shed light into the mechanism of this ‘defection’ under co-infections.

## Materials and Methods

### Measurement of Infectious Units

The concentrations of purified HLSV and TMV virons were measured spectrophotometrically using a Nanodrop spectrophotometer (Nanodrop Technologies) in triplicates, after which serial dilutions were performed (10^−2^, 10^−3^ and 10^−4^ µg/µl). *Chenopodium amaranticolor* plants at 12-leaf stage were inoculated with the diluted HLSV or TMV (in 10 µl) in a Latin square design, followed by 8 h post-inoculation of dark treatment. Plants were grown under 16 h light and 8 h dark at 23°C. The number of local lesions on each inoculated leaves was counted at 6 dpi as an indication of the number of virus infectious unit. Ten ng each of HLSV and TMV provide comparable amount of viral infectious units.

### Virus Inoculation and Sample Collection

Ten ng of HLSV or TMV were inoculated on each chosen leaf of *N. benthamiana* plants at 6–8 leaf stage to establish single infections. For mixed infections, chosen leaves were inoculated with 10 ng each of both viruses in 10 µl inoculation buffer (50 mM KH_2_PO_4_, pH 7.0). Both single and mixed infections were carried out on two new fully expanded leaves per plant. A mock control group at 6–8 leaf stage was treated with the same amount of inoculation buffer. A total of 23 plants were used for each HLSV, TMV, mixed and mock inoculations. The plants were kept at 16 h light and 8 h dark at 23°C until sample collection at 1, 2 and 8 dpi. Half of the infected leaf was cut and stored at −80°C for total RNA extraction. The remaining half of the leaf was left on the plant for observation of symptom expression in order to confirm the infection.

### RNA Extraction and RT–PCR

Total RNAs were extracted from collected *N. benthamiana* leaves as described [27]. The concentration of each RNA sample was measured using Nanodrop2000 (Thermo Scientific) and first-strand cDNA synthesis was performed by using SuperScript™ III Reverse Transcriptase kit (Invitrogen) with 3 µg of total RNA in each reaction system, following manufacturer’s instructions. For single infection, each reaction contained 1 pmol of TMV (5′-AGAGGTCCAAACCAAACCAG-3′) or HLSV (5′- AGCCCAGGATAAACCTGAAG-3′) primer; for mixed infection, both primers were added (1 pmol each). Actin gene was used as an internal control and was amplified using 1 pmol of oligo-dT primer.

### qPCR Assays

All the cDNA samples were subjected to 50 × dilutions for qPCR analysis using BioRad CFX384 Real Time System. Each cDNA was amplified in triplicates each containing 0.5 µl cDNA, 2.5 µl 2 × SYBR Green PCR Master Mix (KAPA), 20 nM of the primers qTMV-F (5′-TAGAGTAGACGACGCAACGG-3′) and qTMV-R (5′-AGAGGTCCAAACCAAACCAG-3′) or qHLSV-F (5′-GAGACTTTGAATGCAACGCA-3′) and qHLSV-R (5′-AGCCCAGGATAAACCTGAAG-3′). All four primers were included for cDNA obtained from mixed infection samples. The actin gene in each sample, which was used as the reference gene, was amplified in triplicates using primers qActin-F (5′-CTTGAAACAGCAAAGACCAGC-3′) and qActin-R (5′-GGAATCTCTCAGCACCAATGG-3′). Relative viral RNA accumulation was determined using 2^−ΔΔCt^ method [28]. Primer efficiency was comparable and repeats were within 2% of each other.

## Supporting Information

Figure S1 Straffin chart for the interactions between Maize chlorotic mottle machlovirus (MCMV) and Wheat streak mosaic rymovirus (WSMV) in N84Ht corn (Scheets, 1998).
(TIF)

Figure S2 Straffin chart for the interactions between Zucchini yellow mosaic virus (ZYMV) and Cucumber mosaic virus (CMV) in zucchini cv. Escandarani (Fattouh, 2003).
(TIF)

Figure S3 Straffin chart for the interactions between Sweet potato chlorotic stunt virus (SPCSV) and Sweet potato feathery mottle virus common strain (SPFMV-C) in sweet potato (Kokkinos & Clark, 2006).
(TIF)

Figure S4 Straffin chart for the interactions between Sweet potato chlorotic stunt virus (SPCSV) and Sweet potato virus G (SPVG) in sweet potato (Kokkinos & Clark, 2006).
(TIF)

Figure S5 Straffin chart for the interactions between Cucumber mosaic virus (CMV) and Pepper mottle virus (PepMoV) in *Capsicum annuum* cv. Early Calwonder (Murphy & Bowen, 2006).
(TIF)

Figure S6 Straffin chart for the interactions between Zucchini yellow mosaic virus (ZYMV-SD) and Cucumber mosaic virus (CMV-Fny) in cucumber (Zeng et al., 2007).
(TIF)

Figure S7 Straffin chart for the interactions between Zucchini yellow mosaic virus (ZYMV-SD) and Cucumber mosaic virus (CMV-Fny) in bottle gourd (Zeng et al., 2007).
(TIF)

Figure S8 Straffin chart for the interactions between Tomato chlorosis virus (ToCV) and Tomato infectious chlorosis virus (TICV) in *Nicotiana benthamiana* (Wintermantel et al., 2008).
(TIF)

Figure S9 Straffin chart for the interactions between Tomato chlorosis virus (ToCV) and Tomato infectious chlorosis virus (TICV) in *Physalis wrightii* (Wintermantel et al., 2008).
(TIF)

Figure S10 Straffin chart for the interactions between Turnip mosaic virus (TuMV) and Cauliflower mosaic caulimovirus (CaMV) in *Arabidopsis thaliana* (Martín & Elena, 2009).
(TIF)

Figure S11 Straffin chart for the interactions between Tomato rugose mosaic virus (ToRMV) and Tomato yellow spot virus (ToYSV) in *Nicotiana benthamiana* (Alves-Junior et al., 2009).
(TIF)

Figure S12 Straffin chart for the interactions between Tomato rugose mosaic virus (ToRMV) and Tomato yellow spot virus (ToYSV) in tomato (Alves-Junior et al., 2009).
(TIF)

Figure S13 Straffin chart for the interactions between Triticum mosaic virus (TriMV) and Wheat streak mosaic virus (WSMV) in wheat cv. Tomahawk (Tatineni et al., 2010).
(TIF)

Figure S14 Straffin chart for the interactions between Triticum mosaic virus (TriMV) and Wheat streak mosaic virus (WSMV) in wheat cv. Arapahoe (Tatineni et al., 2010).
(TIF)
