# Supplementary figures and images for: A Game-Theoretic Model of Interactions between Hibiscus Latent Singapore Virus and Tobacco Mosaic Virus
Source: PLoS One. 2012 May 18;7(5):e37007. doi: 10.1371/journal.pone.0037007 (PMC3356392; doi:10.1371/journal.pone.0037007)

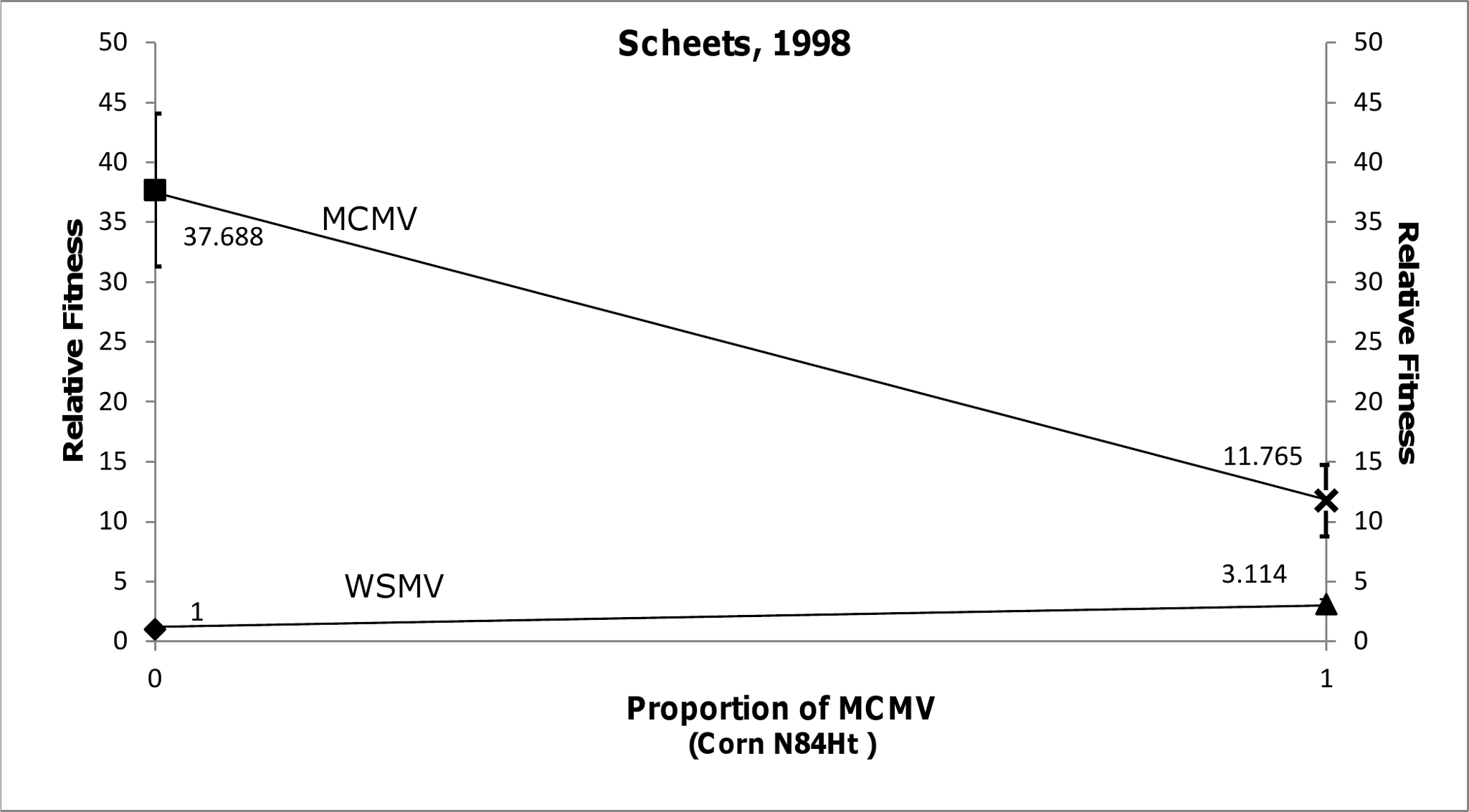

Supplement: Figure S1 — Straffin chart for the interactions between Maize chlorotic mottle machlovirus (MCMV) and Wheat streak mosaic rymovirus (WSMV) in N84Ht corn (Scheets, 1998). (TIF) [file pone.0037007.s001.tif]

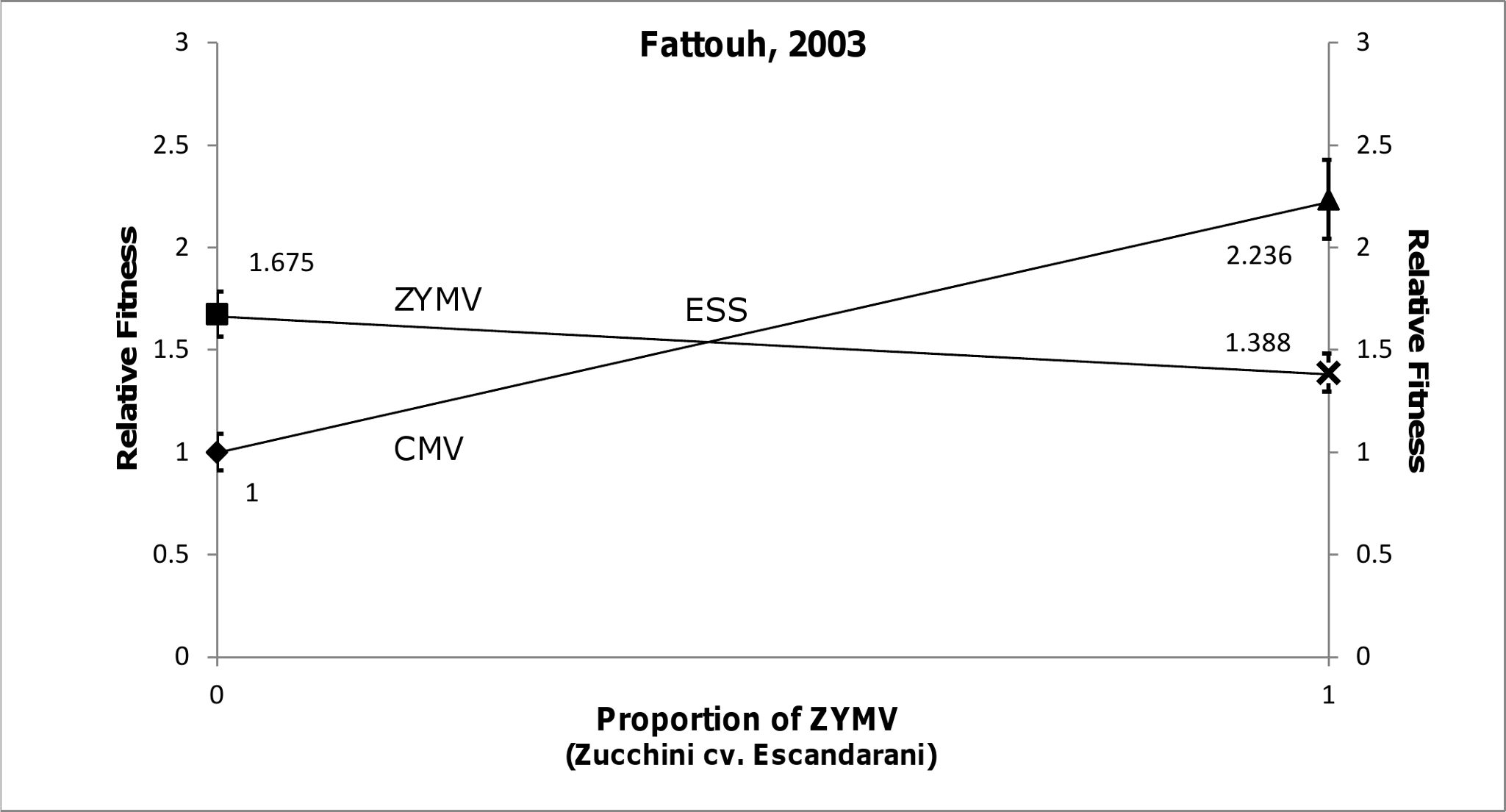

Supplement: Figure S2 — Straffin chart for the interactions between Zucchini yellow mosaic virus (ZYMV) and Cucumber mosaic virus (CMV) in zucchini cv. Escandarani (Fattouh, 2003). (TIF) [file pone.0037007.s002.tif]

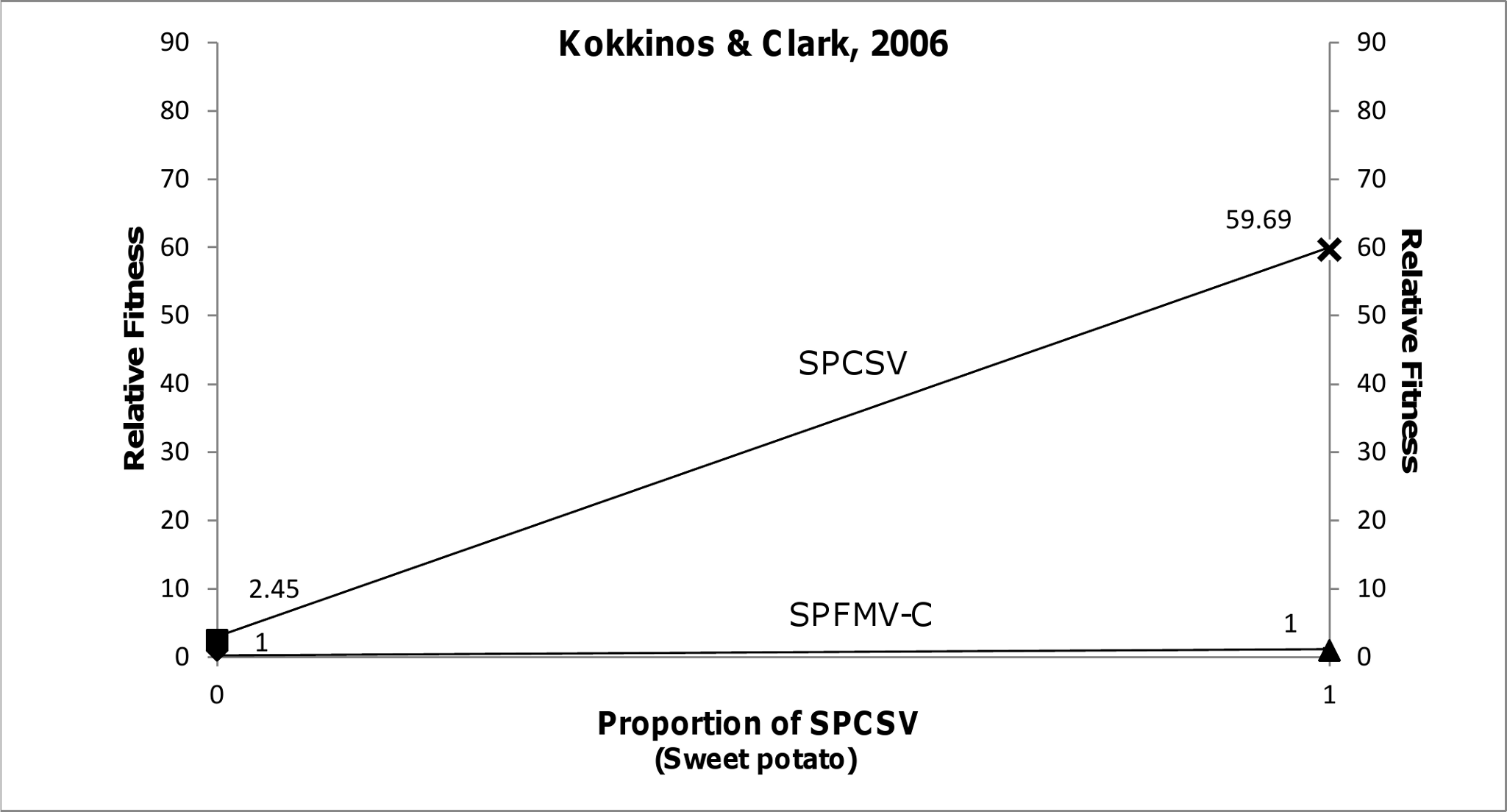

Supplement: Figure S3 — Straffin chart for the interactions between Sweet potato chlorotic stunt virus (SPCSV) and Sweet potato feathery mottle virus common strain (SPFMV-C) in sweet potato (Kokkinos & Clark, 2006). (TIF) [file pone.0037007.s003.tif]

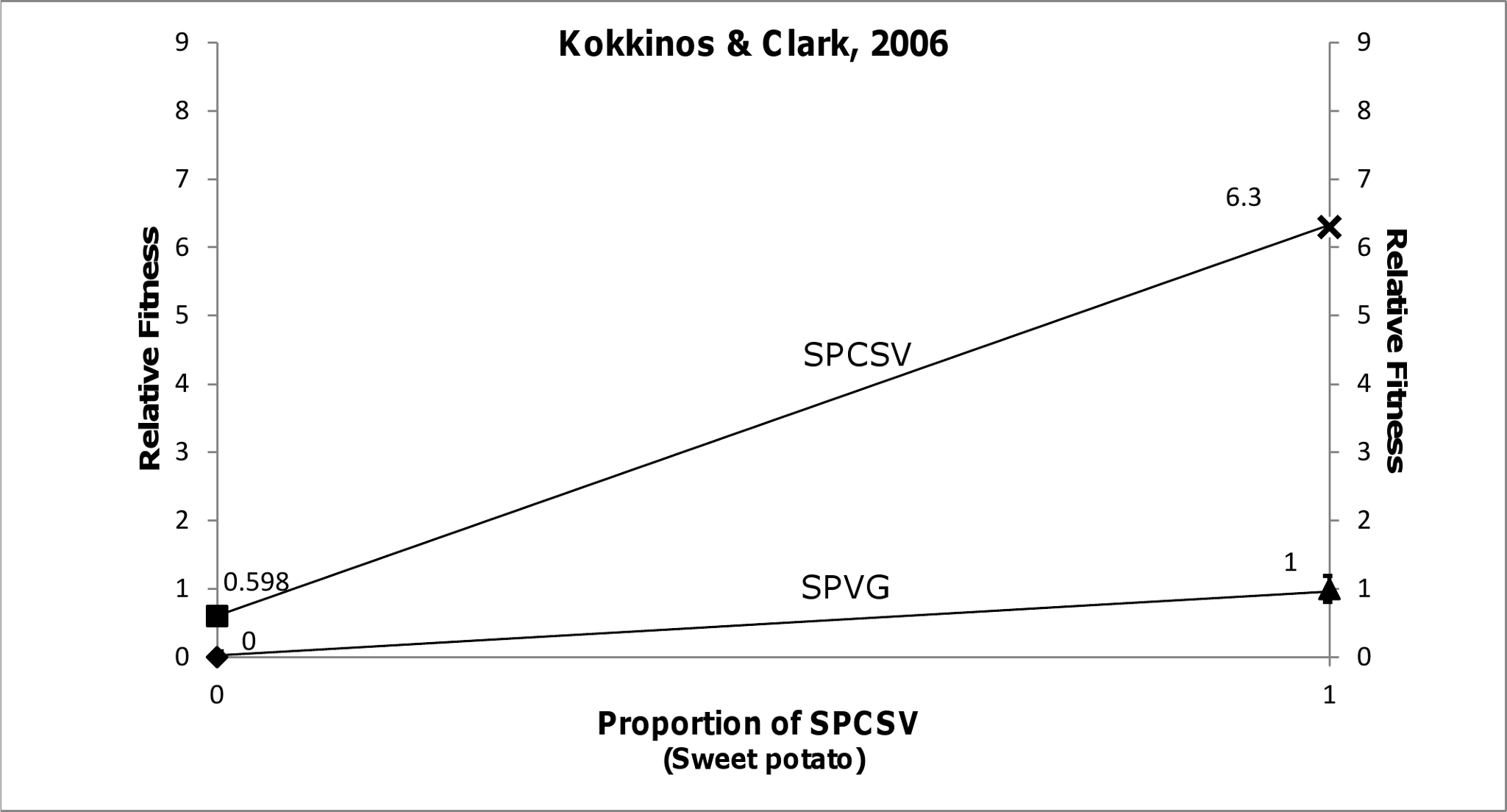

Supplement: Figure S4 — Straffin chart for the interactions between Sweet potato chlorotic stunt virus (SPCSV) and Sweet potato virus G (SPVG) in sweet potato (Kokkinos & Clark, 2006). (TIF) [file pone.0037007.s004.tif]

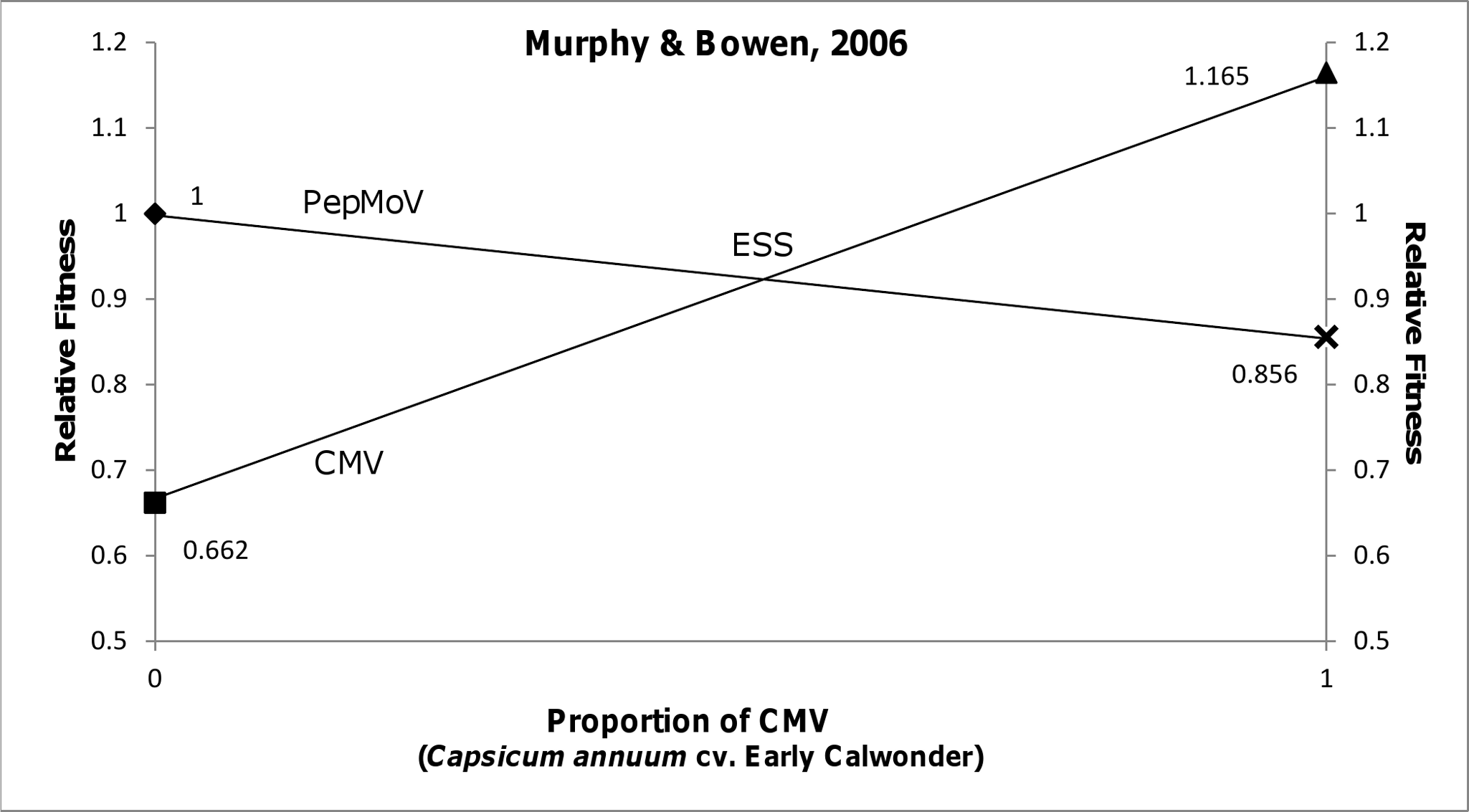

Supplement: Figure S5 — Straffin chart for the interactions between Cucumber mosaic virus (CMV) and Pepper mottle virus (PepMoV) in Capsicum annuum cv. Early Calwonder (Murphy & Bowen, 2006). (TIF) [file pone.0037007.s005.tif]

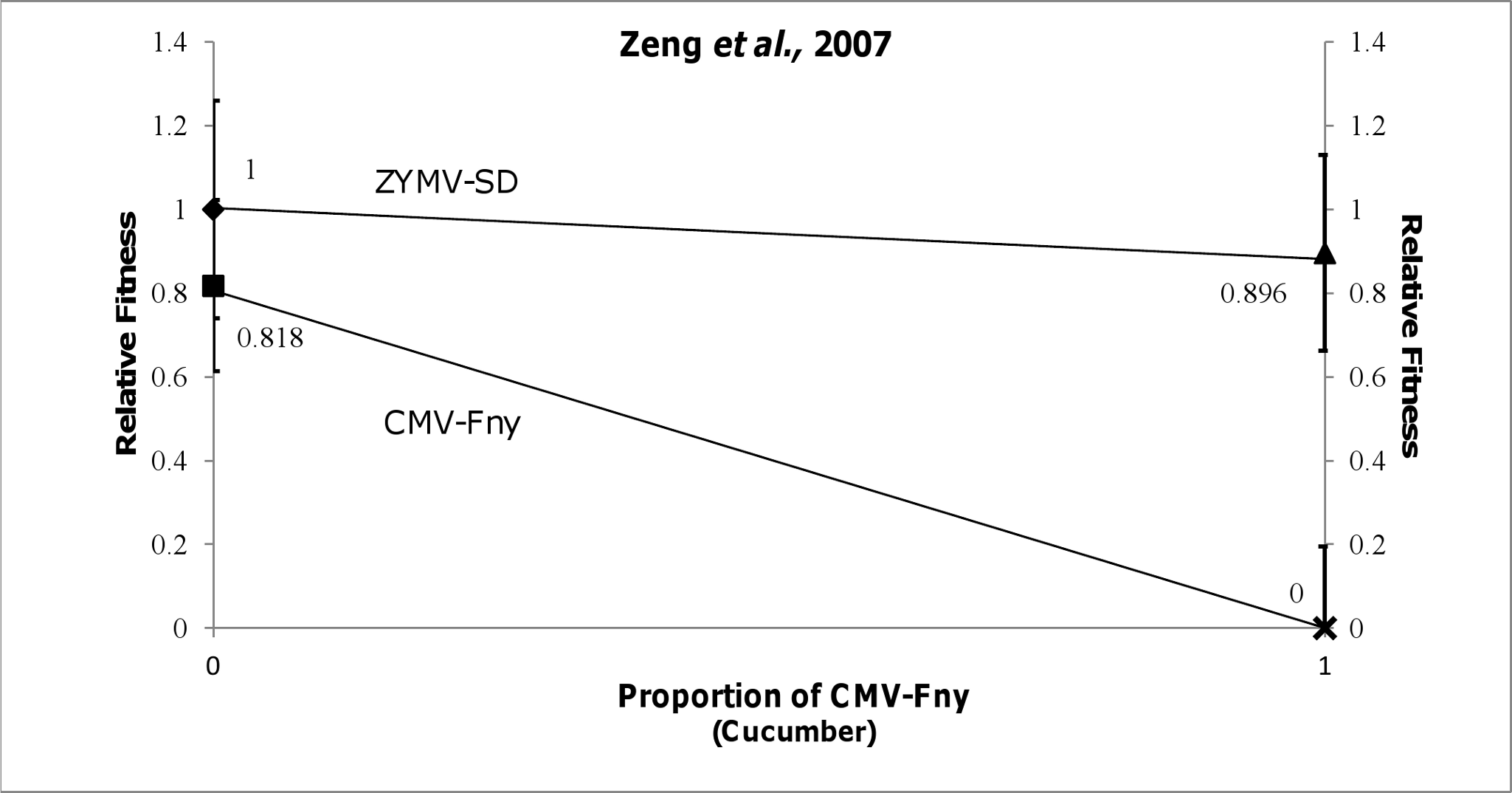

Supplement: Figure S6 — Straffin chart for the interactions between Zucchini yellow mosaic virus (ZYMV-SD) and Cucumber mosaic virus (CMV-Fny) in cucumber (Zeng et al., 2007). (TIF) [file pone.0037007.s006.tif]

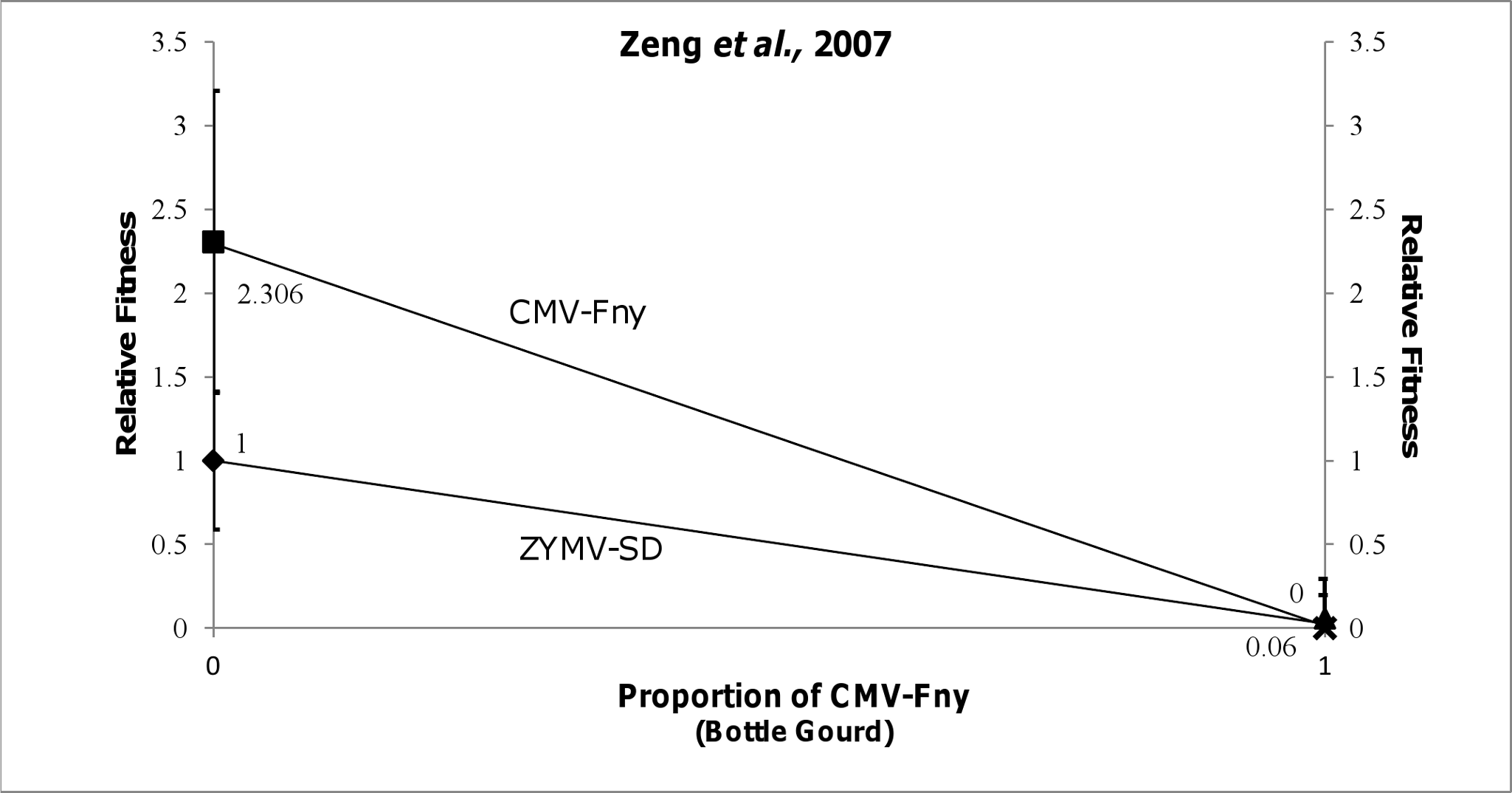

Supplement: Figure S7 — Straffin chart for the interactions between Zucchini yellow mosaic virus (ZYMV-SD) and Cucumber mosaic virus (CMV-Fny) in bottle gourd (Zeng et al., 2007). (TIF) [file pone.0037007.s007.tif]

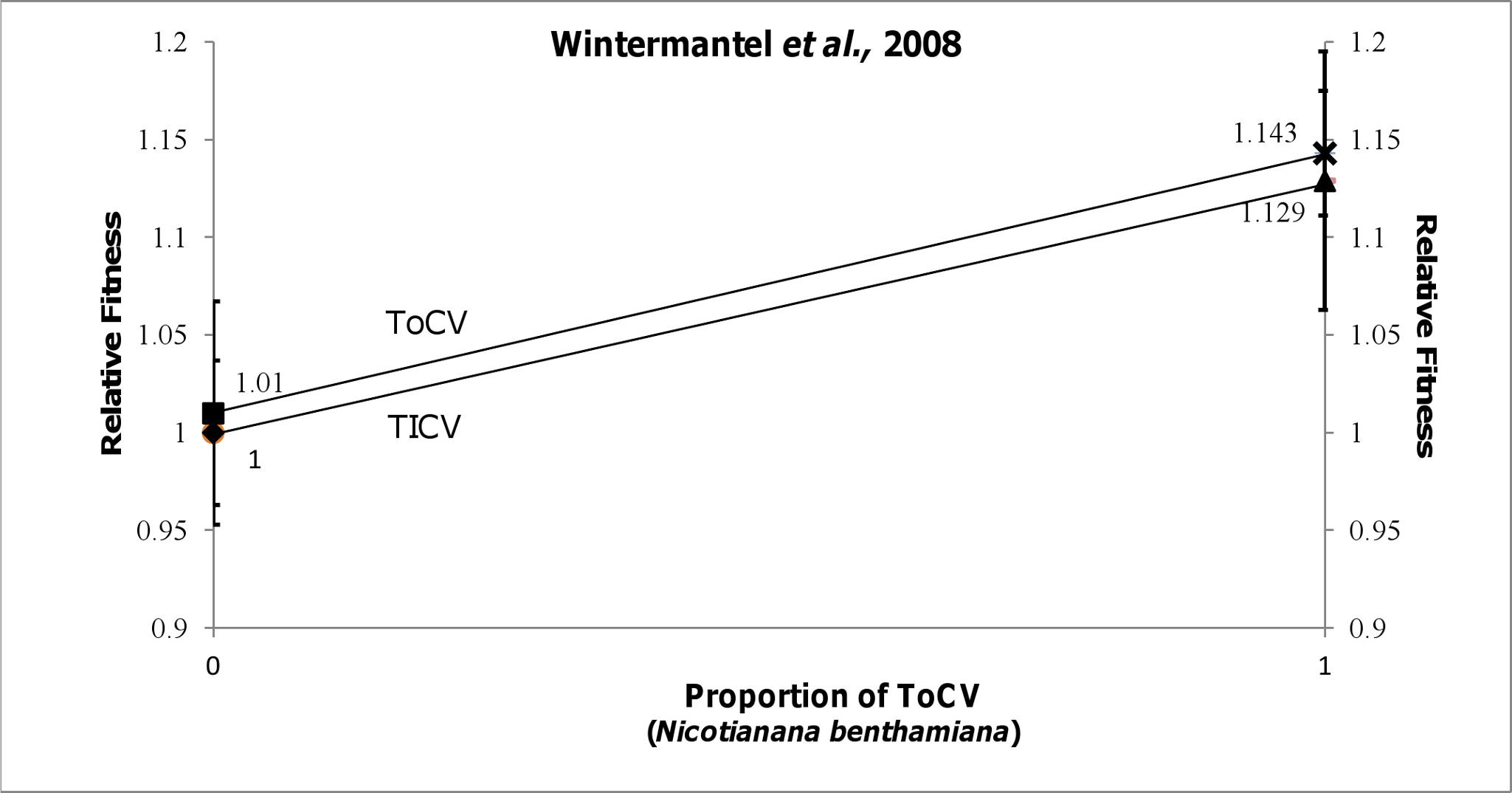

Supplement: Figure S8 — Straffin chart for the interactions between Tomato chlorosis virus (ToCV) and Tomato infectious chlorosis virus (TICV) in Nicotiana benthamiana (Wintermantel et al., 2008). (TIF) [file pone.0037007.s008.tif]

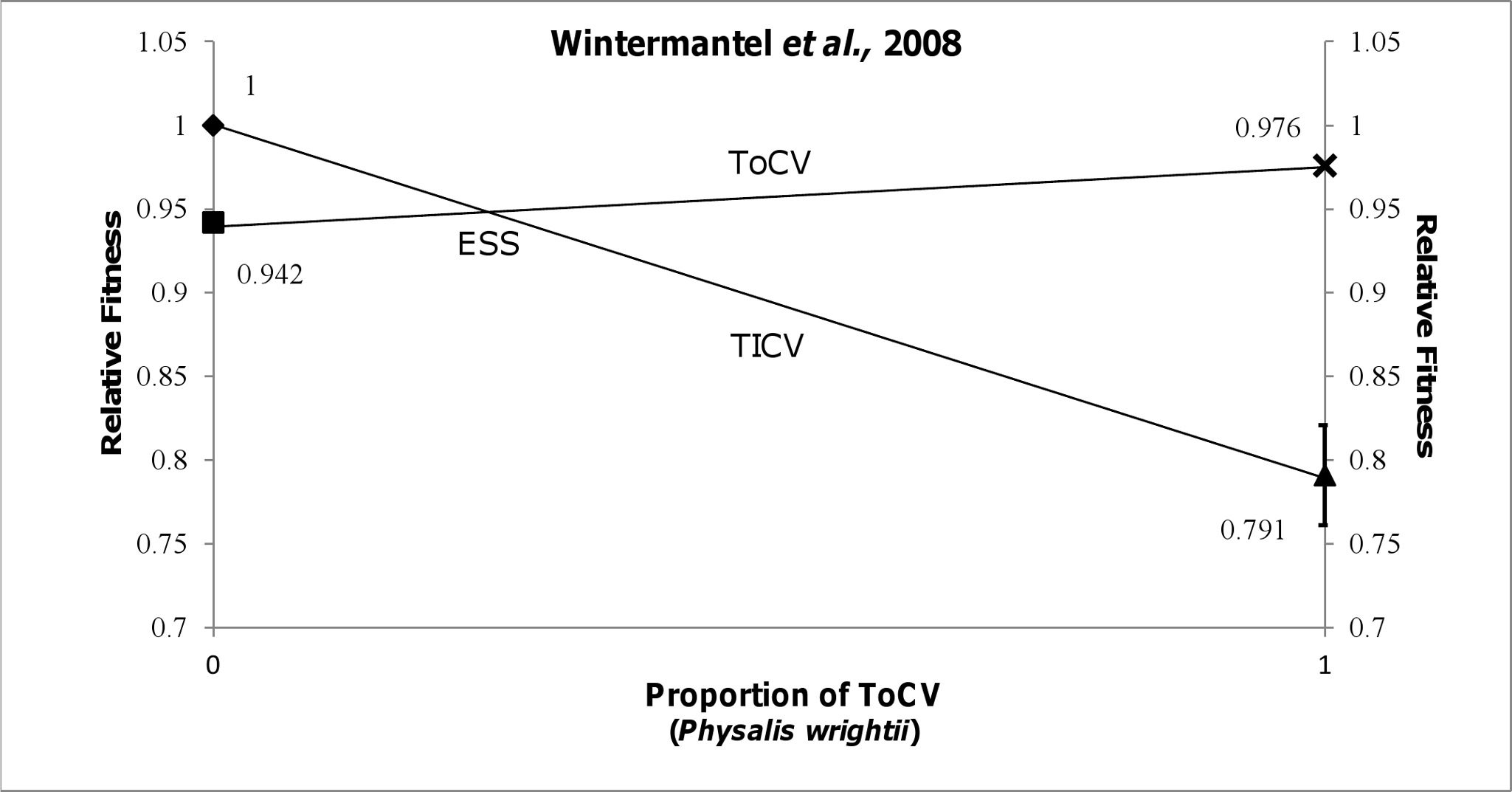

Supplement: Figure S9 — Straffin chart for the interactions between Tomato chlorosis virus (ToCV) and Tomato infectious chlorosis virus (TICV) in Physalis wrightii (Wintermantel et al., 2008). (TIF) [file pone.0037007.s009.tif]

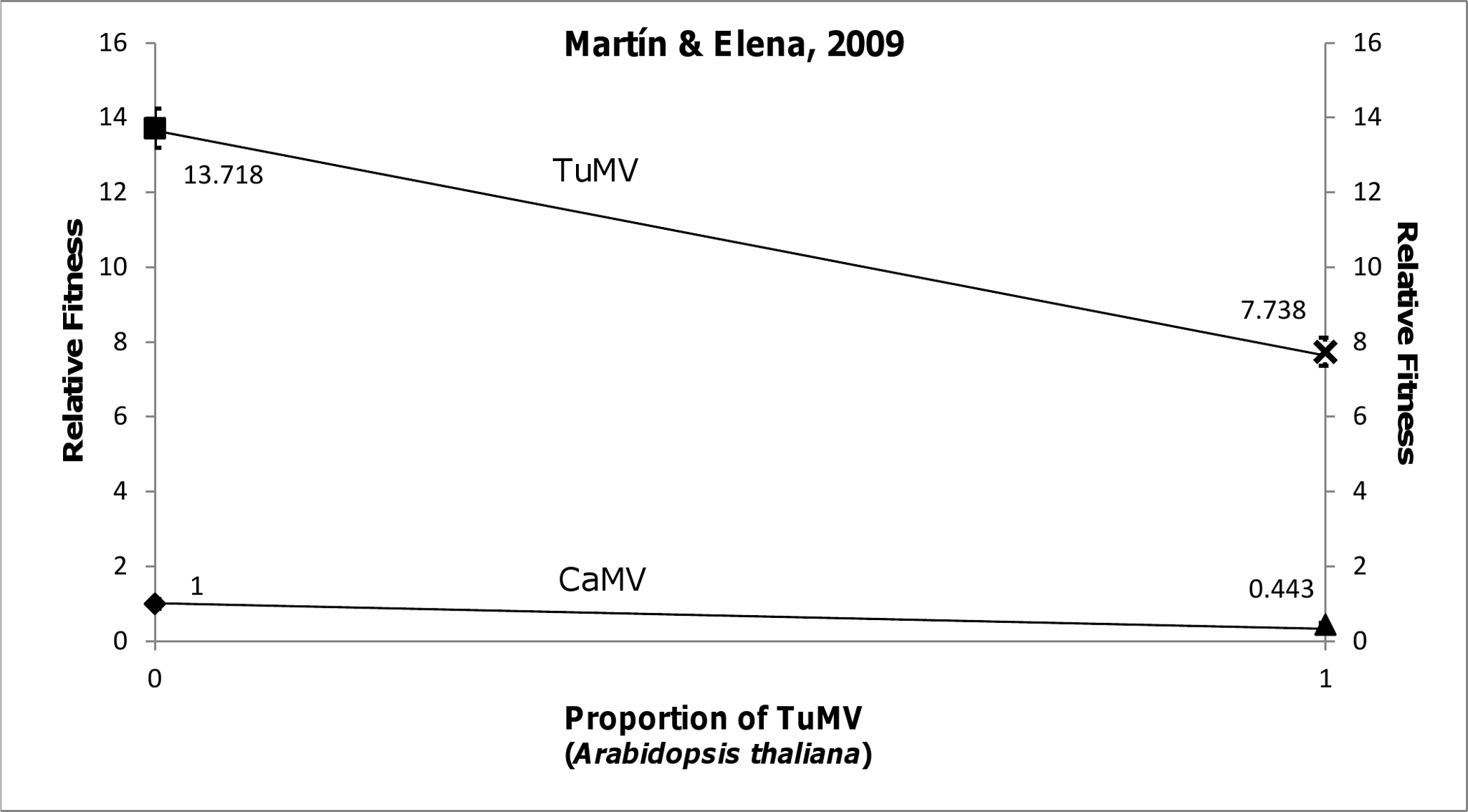

Supplement: Figure S10 — Straffin chart for the interactions between Turnip mosaic virus (TuMV) and Cauliflower mosaic caulimovirus (CaMV) in Arabidopsis thaliana (Martín & Elena, 2009). (TIF) [file pone.0037007.s010.tif]

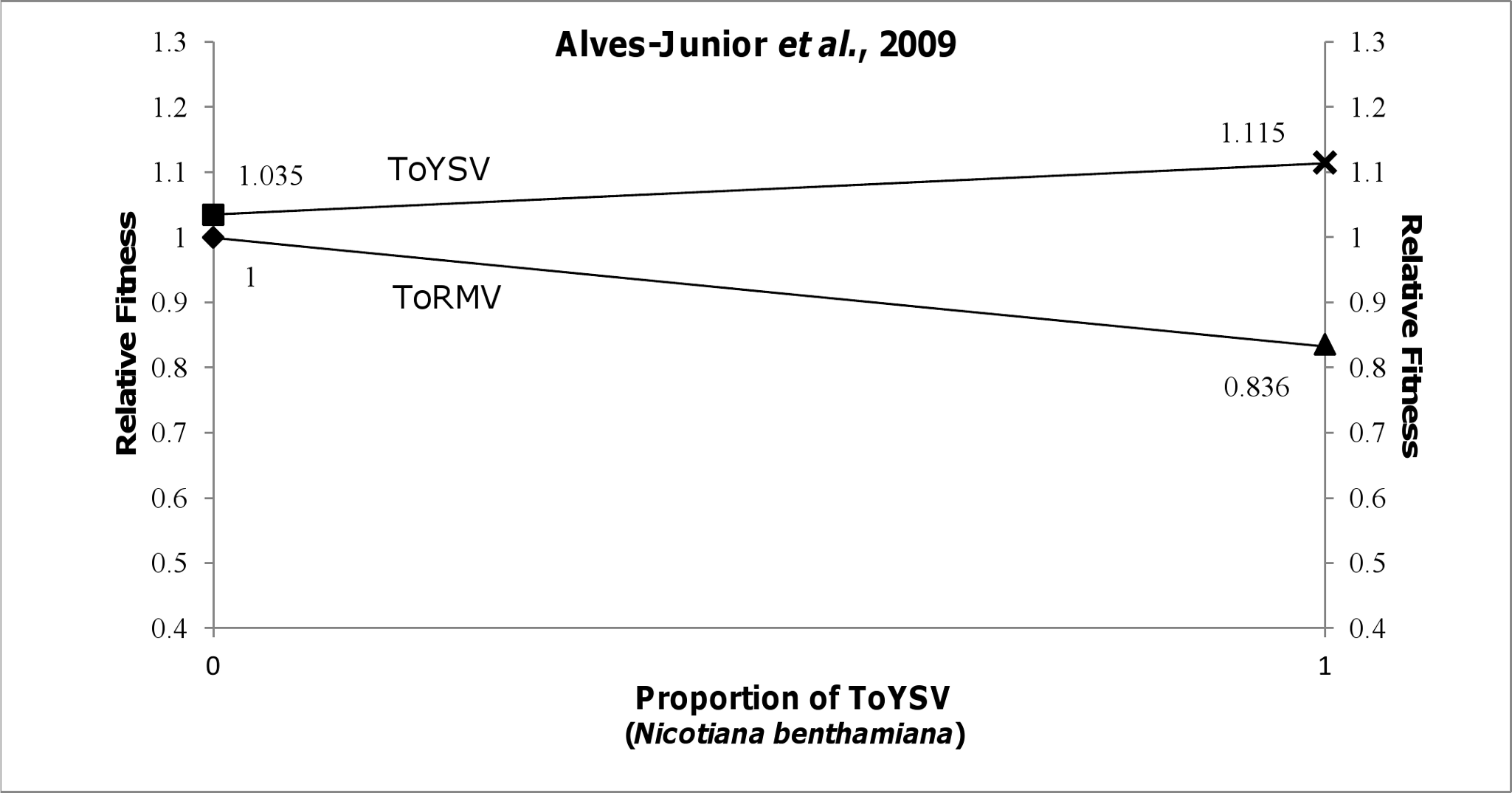

Supplement: Figure S11 — Straffin chart for the interactions between Tomato rugose mosaic virus (ToRMV) and Tomato yellow spot virus (ToYSV) in Nicotiana benthamiana (Alves-Junior et al., 2009). (TIF) [file pone.0037007.s011.tif]

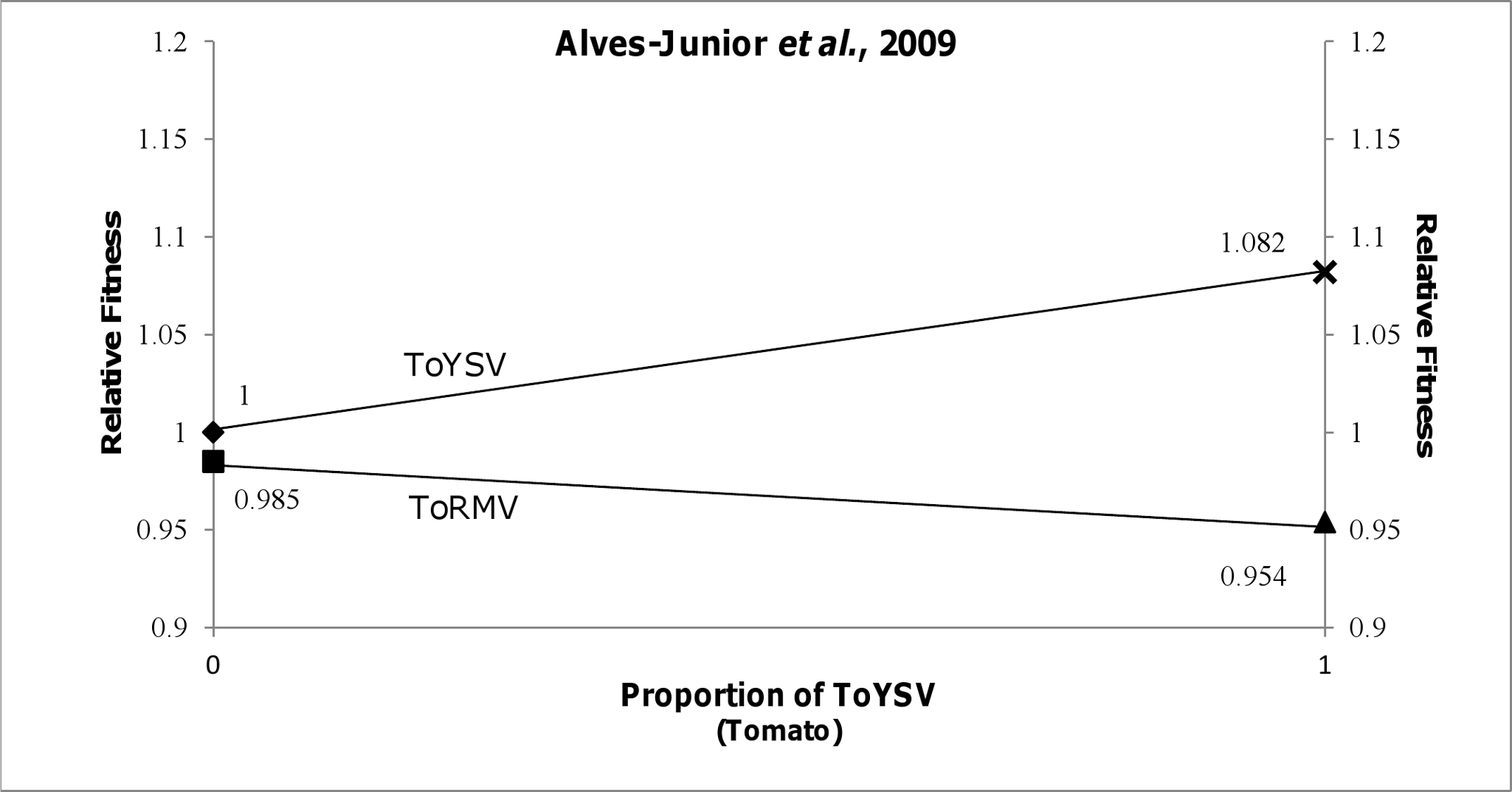

Supplement: Figure S12 — Straffin chart for the interactions between Tomato rugose mosaic virus (ToRMV) and Tomato yellow spot virus (ToYSV) in tomato (Alves-Junior et al., 2009). (TIF) [file pone.0037007.s012.tif]

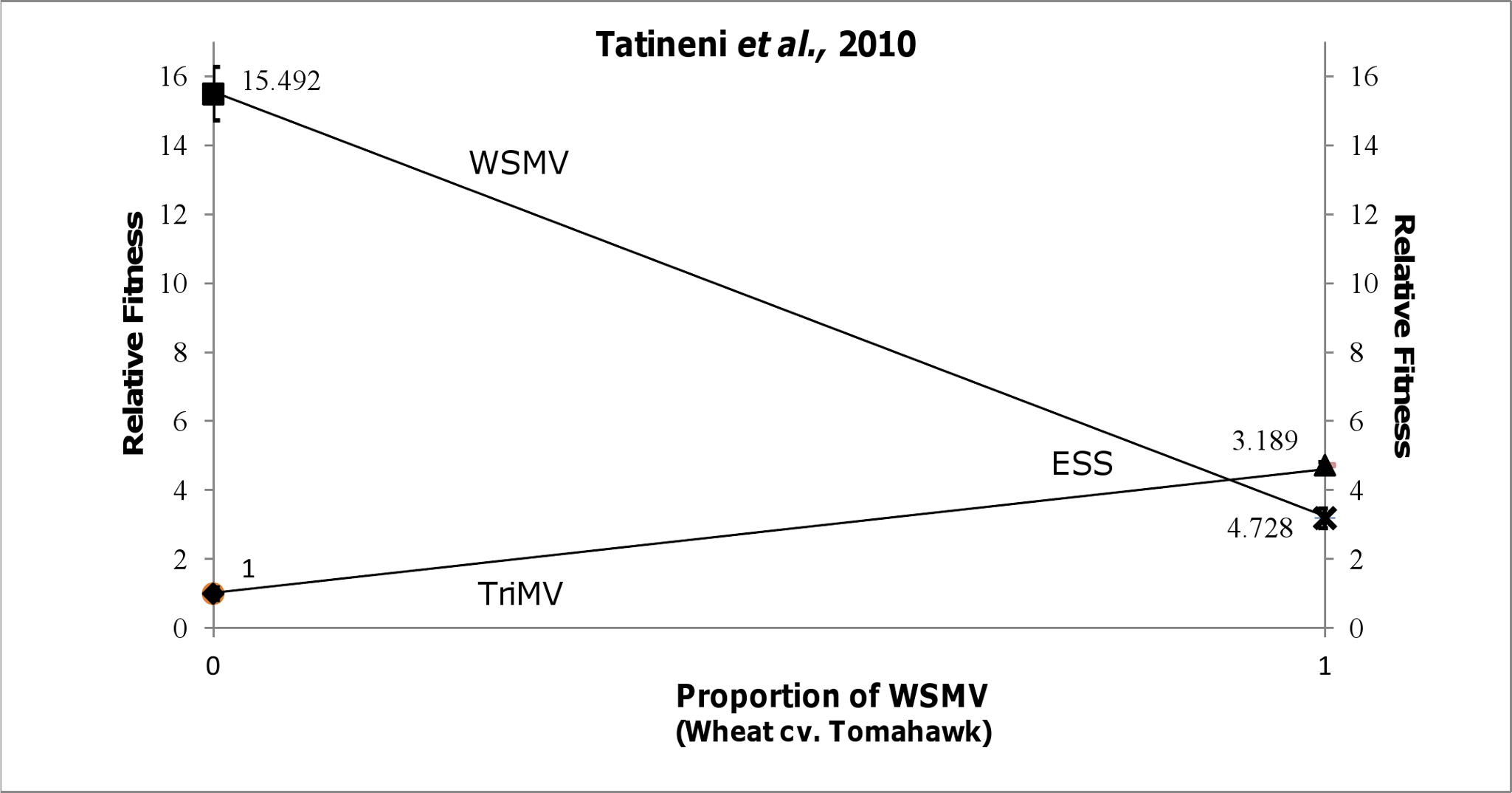

Supplement: Figure S13 — Straffin chart for the interactions between Triticum mosaic virus (TriMV) and Wheat streak mosaic virus (WSMV) in wheat cv. Tomahawk (Tatineni et al., 2010). (TIF) [file pone.0037007.s013.tif]

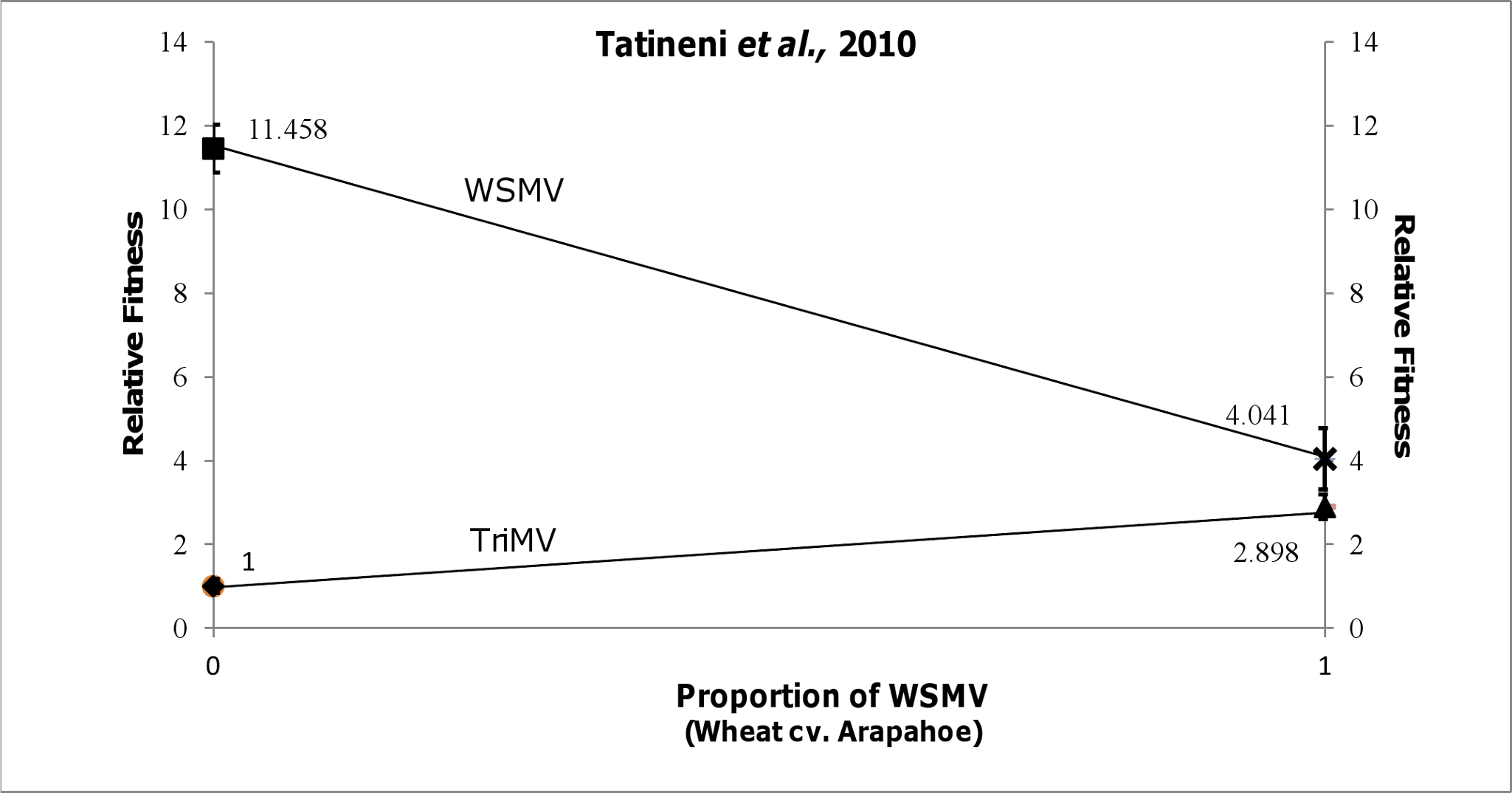

Supplement: Figure S14 — Straffin chart for the interactions between Triticum mosaic virus (TriMV) and Wheat streak mosaic virus (WSMV) in wheat cv. Arapahoe (Tatineni et al., 2010). (TIF) [file pone.0037007.s014.tif]
